# Altered endotoxin responsiveness in healthy children with Down syndrome

**DOI:** 10.1186/s12865-018-0270-z

**Published:** 2018-11-03

**Authors:** Dean Huggard, Fiona McGrane, Niamh Lagan, Edna Roche, Joanne Balfe, Timothy Ronan Leahy, Orla Franklin, Ana Moreno, Ashanty M. Melo, Derek G. Doherty, Eleanor J. Molloy

**Affiliations:** 1 Paediatrics, Trinity College, the University of Dublin, Dublin, Ireland; 2 Trinity Translational Medicine Institute (TTMI), Trinity College Dublin, Dublin, Ireland; 3 Paediatrics, Tallaght Hospital, Dublin, Ireland; 4 Coombe Women and Infants University Hospital, Dublin, Ireland; 5 Neonatology, Our Lady’s Children’s Hospital, Crumlin, Dublin, Ireland; 6 Immunology, Our Lady’s Children’s Hospital, Crumlin, Dublin, Ireland; 7 Cardiology, Our Lady’s Children’s Hospital, Crumlin, Dublin, Ireland; 8 National Children’s Research Centre, Our Lady’s Children’s Hospital, Crumlin, Dublin, Ireland; 9 Department of Paediatrics, Trinity Centre for Health Sciences, Tallaght Hospital, Dublin, 24 Ireland

**Keywords:** Down syndrome, Inflammation, Endotoxin, Innate immunity, Immunomodulation

## Abstract

**Background:**

Down syndrome (DS) is the most common syndromic immunodeficiency with an increased risk of infection, mortality from sepsis, and autoinflammation. Innate immune function is altered in DS and therefore we examined responses in CD11b and Toll like receptor 4 (TLR-4), which are important immune cell surface markers upregulated in response to Lipopolysaccharide (LPS) endotoxin, and the immunomodulator melatonin. Neutrophil and monocyte responses to LPS and melatonin in children with Down syndrome (DS) who were clinically stable were compared to age-matched controls. Whole blood was incubated with LPS and melatonin and the relative expression of CD11b and TLR-4 evaluated by flow cytometry.

**Results:**

Children with DS had an increased response to LPS in neutrophils and intermediate monocytes, while also having elevated TLR-4 expression on non-classical monocytes compared to controls at baseline. Melatonin reduced CD11b expression on neutrophils, total monocytes, both classical and intermediate sub-types, in children with DS and controls.

**Conclusion:**

Melatonin could represent a useful clinical adjunct in the treatment of sepsis as an immunomodulator. Children with DS had increased LPS responses which may contribute to the more adverse outcomes seen in sepsis.

## Background

Down syndrome (DS) is caused by an extra copy of genetic material from chromosome 21, and is the most prevalent chromosomal abnormality, affecting approximately 1 in 550 births in Ireland [1], and 1 in 700 births in the USA [2]. Co-morbidities associated with DS include developmental disability, congenital heart disease (CHD), gastrointestinal tract anomalies, and an increased risk of haematological malignancy [3]. In addition, it is the most common genetic syndrome associated with abnormal immune function and immune defects [4]. There is significant evidence of immune dysregulation in Down syndrome including T-cell and B-cell lymphopenia due to impaired expansion of these cell lines in infancy [5], a smaller thymus gland with reduced naïve Tcell and regulatory T-cell numbers [6], suboptimal antibody responses to vaccination [7–10], and abnormal levels of serum cytokines [11–13].

Children with Down syndrome are, therefore, at increased risk of infection, especially in early childhood, particularly respiratory tract infections [14]. Hilton et al. [15] reported a higher risk of admission to hospital and intensive care with respiratory tract infections (RTIs) in children with DS. Mortality from sepsis is 30% greater in patients with DS in comparison to children without DS who also had sepsis [16].

It is challenging to attribute causation to a specific deficit of the immune system with the increased incidence of infections and sepsis seen in this cohort. A normal innate immune system is crucial in providing first line defence against infection. Neutrophils and monocytes are crucial cellular components of the innate immune system. Defective phagocytic activity and neutrophil chemotaxis have previously been reported in DS [17, 18]. Monocyte function in DS is poorly described. Increased numbers of the non-classical (CD14dim/CD16+) monocyte pro-inflammatory sub-type have been described in DS in comparison to controls [19]. This monocyte population has previously been implicated in sepsis and chronic disease [20].

CD11b is a cell surface marker involved in mediating neutrophil and monocyte adhesion and diapedesis [21] and is an indicator of activation. Dysfunction in neutrophil adherence and migration has been shown to increase the risk of infection in adults and neonates [22]. Toll-like receptor 4 (TLR-4) is the key receptor involved in lipopolysaccharide (LPS) endotoxin recognition and activation of the innate immune system [23], and has also been implicated in the pathogenesis of autoimmune conditions such as systemic lupus erythematosus (SLE) and rheumatoid arthritis [24].

Immunomodulators can alter responses to infection, alleviate autoimmunity and ultimately improve patient care. Melatonin is an endogenous hormone which mediates its anti-inflammatory effects by modulating pro-inflammatory cytokines and inflammasome de-activation, thereby ameliorating results in endotoxaemia [25, 26]. Melatonin has a very good safety profile and is used in paediatrics in sleep management [27]. Clinical trials in adults and neonates with sepsis have demonstrated improved clinical outcomes [28, 29].

We hypothesized that children with DS have altered neutrophil and monocyte function which contributes to their increased susceptibility to infection and increased mortality from sepsis. We aimed to evaluate the in vitro effect of LPS endotoxin, and the anti-inflammatory melatonin on CD11b and TLR4 expression on neutrophils and monocytes in children with DS.

## Methods

### Study population

This study was approved by the ethics committees in the National Children’s Hospital, Tallaght and Our Lady’s Children’s Hospital, Crumlin (OLCHC), Dublin, Ireland. All parents and participants received verbal and written information on the study and written consent was obtained in advance of recruitment. There were two patient groups studied: a) Healthy children with Down syndrome < 16 years old attending the dedicated Down syndrome clinic. All children were clinically well with no recent fever or evidence infection and were undergoing annual routine health surveillance and b) Age-matched Controls: healthy controls attending phlebotomy or for day case procedures. Blood sampling occurred at induction of general anaesthetic and controls had no recent fever or evidence of infection.

### Experimental design

All blood samples (1-3 mL) for in vitro experiments were collected in a sodium citrate anti-coagulated blood tube and analysed within 2 h of sample acquisition. Blood sampling coincided with routine phlebotomy or at induction of anaesthesia for day case procedures. Whole blood was incubated at 37 °C for 1 h with the pro-inflammatory stimulant Lipopolysaccharide (LPS; *E.coli* 0111:B4: SIGMA Life Science, Wicklow, Ireland) 10 ng/mL, the anti-inflammatory agent Melatonin (SIGMA Life Science, Wicklow, Ireland) at 42 *μ*M and both combined.

Blood samples were incubated with a dead cell stain (100 μL; (Fixable Viability Dye eFlour 506, Invitrogen, California USA), diluted to working concentration in phosphate buffered saline (PBS). The following fluorochrome-labelled monoclonal antibodies (mAb) were added to each sample (2.5 μL per tube): CD14-PerCP, CD15-PECy7, CD16-FITC, CD66b-Pacific Blue and TLR4-APC (BioLegend®, California, USA) and PE labelled CD11b (BD Biosciences, Oxford, UK; 10 μL per tube. PBA buffer (PBS containing 1% bovine serum albumin and 0.02% sodium azide) was used to make up the antibody cocktail. Samples were incubated in the dark for 15 min. Next 1 mL of FACS lysing solution (BD Biosciences, Oxford, UK) was added to each tube, the samples were then incubated for 15 min in the dark. Cells were pelleted by centrifugation at 450 g for 7 min at room temperature, washed twice with PBA buffer and fixed in 300 μL of 1% paraformaldehyde. The final cell pellet was resuspended in 100 μL PBA buffer and analysed on a BD FACS Canto II flow cytometer.

### Quantification of cell surface antigen expression

**Fig. 1:**
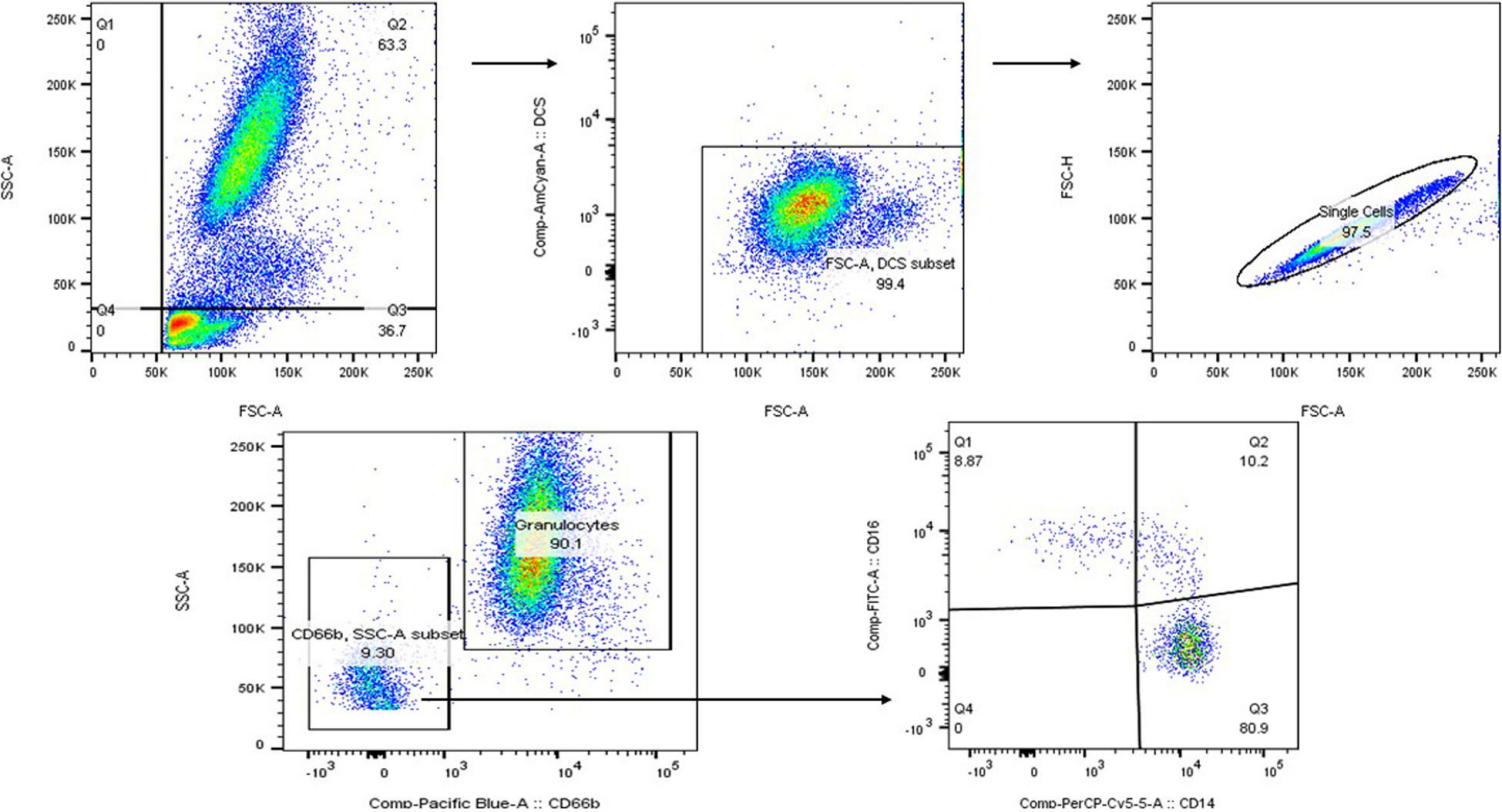
Gating strategy for isolation of granulocytes and monocyte sub-populations. Neutrophils were delineated based on SSC-A and CD66b + positivity. Monocytes were defined based on SSC-A, CD66b- and subsets based on relative CD14+ CD16+ populations; classical (CD14+/CD16-), intermediate (CD14+/CD16+), non-classical (CD14dim/CD16+)

The expression of CD11b and TLR-4 antigens on the surface of neutrophils and monocytes was evaluated by flow cytometry on the BD FACS Canto II cytometer. Neutrophils were delineated based on SSC-A and CD66b + positivity as previously described [30], monocytes were defined based on SSC-A, CD66b- and subsets based on relative CD14+ CD16+ populations; classical (CD14+/CD16-), intermediate (CD14+/CD16+), non-classical (CD14dim/CD16+), Fig. 1. A minimum of 10,000 events were collated and relative expression of CD11b and TLR-4 was expressed as mean channel fluorescence (MFI), and analysed using FloJo software (Oregon, USA). Every sample was processed and analysed by the same researcher (DH) thereby reducing variability in results.

### Statistics

Statistical analysis was done using paired and un-paired *t* tests to compare mean results between two independent cohorts. Significance was defined as *p* < 0.05. Results shown are expressed as mean +/− standard error of the mean (SEM) unless otherwise stated. Data was analysed with FloJo software (Oregon, USA) and GraphPad Prism.

## Results

### Patient characteristics

There were 23 healthy children with Down syndrome (DS) with a mean ±SD age of 8.67 ± 4 years(y) of which 13 were female (57%), and 21 healthy controls with a mean age of 7.4 ± 4.60 y, of which 10 were female (48%). In the DS cohort, children with a history of significant congenital heart disease requiring surgery in infancy (*n* = 7) were all clinically stable with no further cardiology intervention. All control participants had no significant medical history. Both groups were well at the time of blood sampling with no recent history of infection.

### Effects of LPS endotoxin on CD11b expression

Neutrophil baseline CD11b expression in children with DS was significantly lower compared with controls (*p* = 0.045). Following incubation with LPS, CD11b significantly increased in both groups (Fig. 1a: DS *p* < 0.0001; Control *p* = 0.0001). When comparing the fold increase in CD11b expression from baseline, children with DS had a significantly higher rise after LPS stimulation (DS: Controls: 116% versus 62.4%; *p* = 0.03; Fig. 3a).

**Fig. 2:**
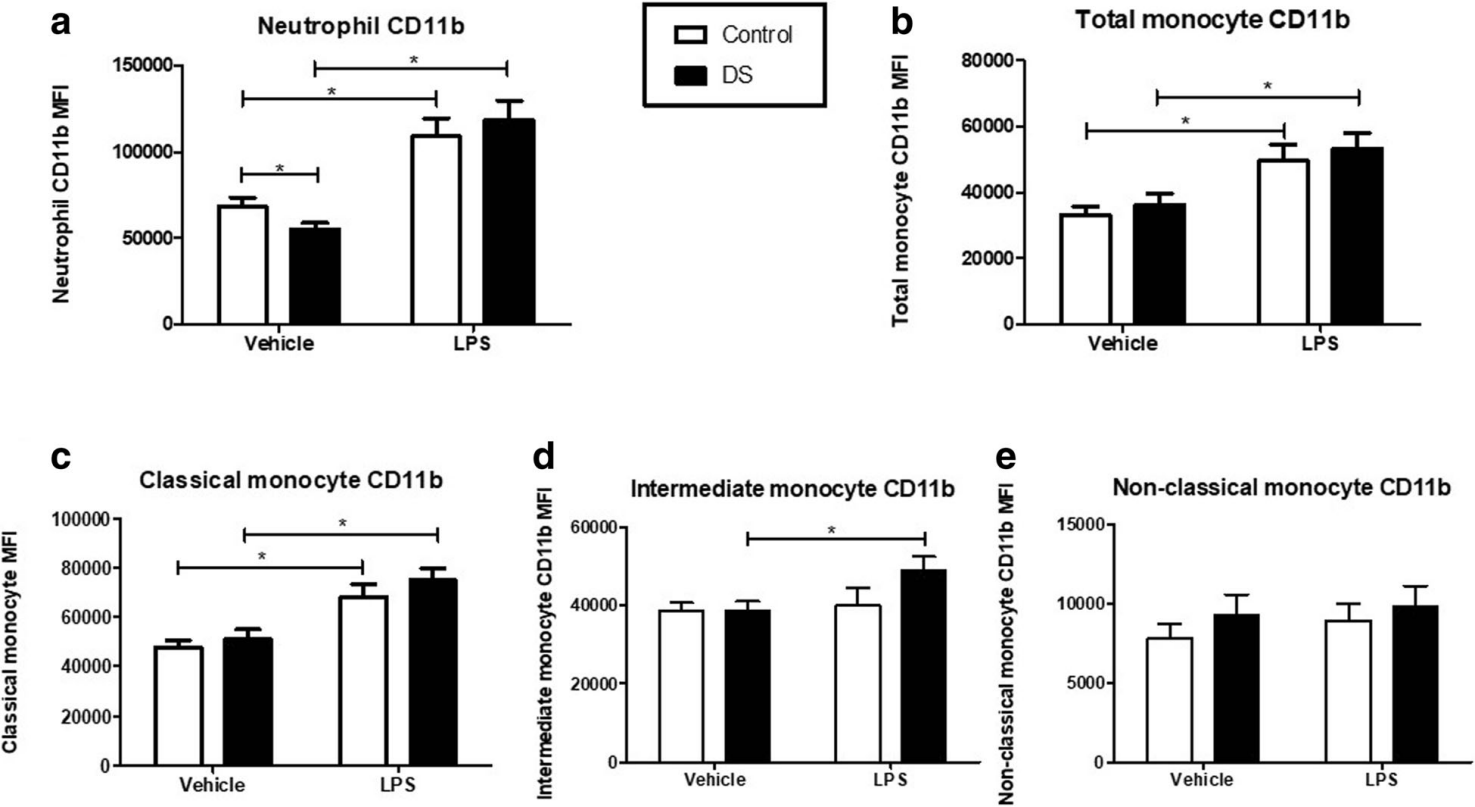
Neutrophil and monocyte CD11b expression in response to lipopolysaccharide (LPS) in children with Down syndrome (DS) and controls. Values expressed as mean channel fluorescence (MFI). *p < 0.05. **a** Neutrophil CD11b (DS *n* = 23; Controls *n* = 16); **b** Total monocyte CD11b (DS *n* = 19; Controls *n* = 21); **c** Classical monocyte CD11b (DS *n* = 19; Controls *n* = 21; **d** Intermediate monocyte CD11b (DS *n* = 18; Controls *n* = 20); **e** Non-classical monocyte (DS *n* = 19; Controls *n* = 21)

CD11b expression on total monocytes showed no difference at baseline or after LPS stimulation between both groups (Fig. 2b *p* = 0.48). The percentage rise of CD11b expression after LPS was similar in both groups also (DS versus Control: 53 v 55%; *p* = 0.92 Fig. 4b). Monocyte subset CD11b expression analysis revealed no significant differences at baseline or after LPS stimulation in classical (CD14+/CD16-) (*p* = 0.74), and non-classical (CD14dim/CD16+) (*p* = 0.21) sub-populations in children with DS versus controls (Figs. 2c, e and 4c (*p* = 0.55), e (*p* = 0.56)). Intermediate monocytes (CD14+/CD16+) demonstrated no difference in CD11b expression at baseline in children with DS and controls (*p* = 0.87). After LPS stimulation there was a significant increase in CD11b in children with DS (*p* = 0.004) but not in controls (*p* = 0.78; Fig. 2d). The mean percentage rise in CD11b expression in children with DS was not significantly increased compared to controls (DS vs controls: 31.8 v 5.8%; *p* = 0.088; Fig. 4d).

Classical monocytes (CD14+/CD16-) exhibited the highest CD11b expression at baseline compared with the other sub-populations in both cohorts (DS vs Control -classical vs intermediate: *p* = 0.009 v 0.01). This sub-population also demonstrated the largest mean percentage rise in CD11b after LPS treatment in both children with DS and controls. (DS – classical % rise vs intermediate vs nonclassical = 52.1 vs 31.8 vs 15.3%; Controls - 44 vs 5.8 vs 24%). Non-classical monocytes (CD14dim/CD16+) demonstrated the lowest mean CD11b expression at baseline of any sub-population, in both children with DS and controls. This was significantly lower compared to both intermediate and classical monocyte CD11b in both cohorts.

### Effects of LPS endotoxin on TLR4 expression

**Fig. 3:**
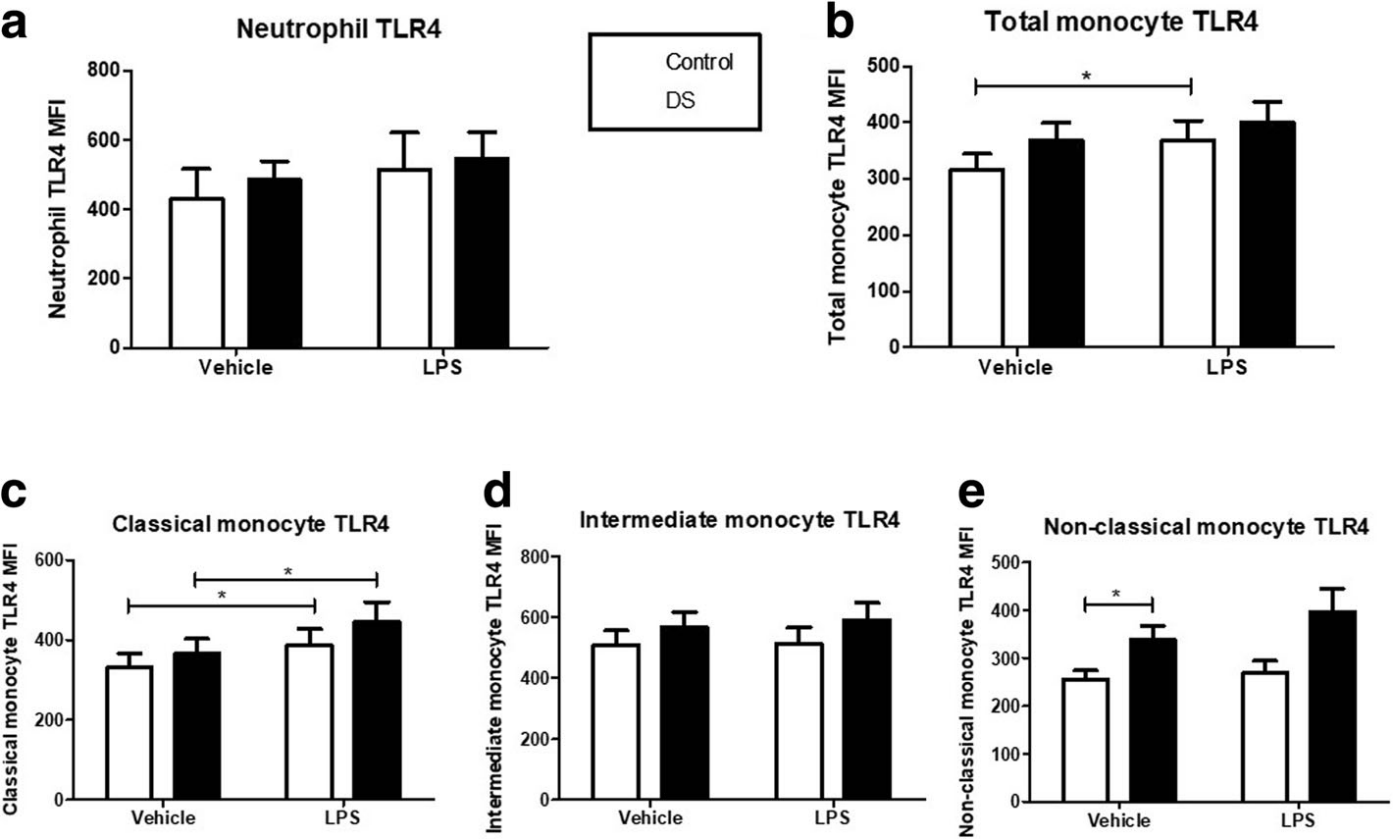
Neutrophil and monocyte Toll-like receptor (TLR-4) expression in response to lipopolysaccharide (LPS) in children with Down syndrome (DS) and controls. Values expressed as mean channel fluorescence (MFI). *p < 0.05. **a** Neutrophil TLR4 (DS *n* = 19; Controls *n* = 10); **b** Total monocyte TLR-4 (DS *n* = 22; Controls *n* = 15); **c** Classical monocyte TLR4 (DS *n* = 16; Controls *n* = 15); **d** Intermediate monocyte TLR4 (DS *n* = 15; Controls *n* = 14); **e** Non-classical monocyte TLR-4 (DS *n* = 16; Controls *n* = 20)

Neutrophil TLR-4 expression at baseline was not significantly different between children with DS compared to controls (*p* = 0.57). After LPS incubation there was no significant response in TLR4 expression in either cohort (DS *p* = 0.15 v Control *p* = 0.057; Fig. 3a). On comparing the mean percentage rise in TLR-4 expression after LPS, there was a 9.4% rise in children with DS versus 28.7% in the control group (Fig. 5a (*p* = 0.23)).

TLR-4 expression on total monocytes did not show any difference at baseline between children with DS and controls (*p* = 0.24). TLR-4 expression post LPS treatment increased significantly in controls (*p* = 0.016) but did not reach significance in the children with DS (*p* = 0.07; Fig. 3b). The mean percentage rise after LPS stimulation was 8.4% in children with DS versus 17.2% in controls ((*p* = 0.2)) Fig. 5b). Monocyte subset TLR-4 expression analysis revealed no significant differences at baseline or after LPS treatment in classical (CD14+/CD16-) or intermediate (CD14+/CD16+) subpopulations between children with DS and controls (Fig. 3c (*p* = 0.51), d (*p* = 0.4) and Fig. 5c (*p* = 0.75), d (*p* = 0.84)). Non-classical monocyte (CD14dim/CD16+) TLR-4 expression was found to be significantly higher at baseline in children with DS compared to controls (*p* = 0.02; Fig. 3e). There were no significant differences in TLR-4 expression after LPS stimulation in either cases or controls (Fig. 5e (*p* = 0.96)).

The classical monocytes in both cohorts exhibited the largest mean percentage rise in TLR-4 expression after LPS treatment (DS – classical vs intermediate vs non-classical = 15.2 vs 3.6 vs 9.9%; Control - 17.9 vs 1.2 vs 10.7%). Intermediate monocytes had the largest mean TLR-4 MFI at baseline of any monocyte subpopulation in both children with DS and the control group (DS v control: intermediate vs classical *p* = 0.003 versus 0.005). Non-classical monocytes displayed the lowest mean TLR-4 at baseline of the three monocyte subsets and was significantly lower than intermediate monocyte TLR-4 in both cohorts.

### Effects of melatonin on CD11b expression

**Fig. 4:**
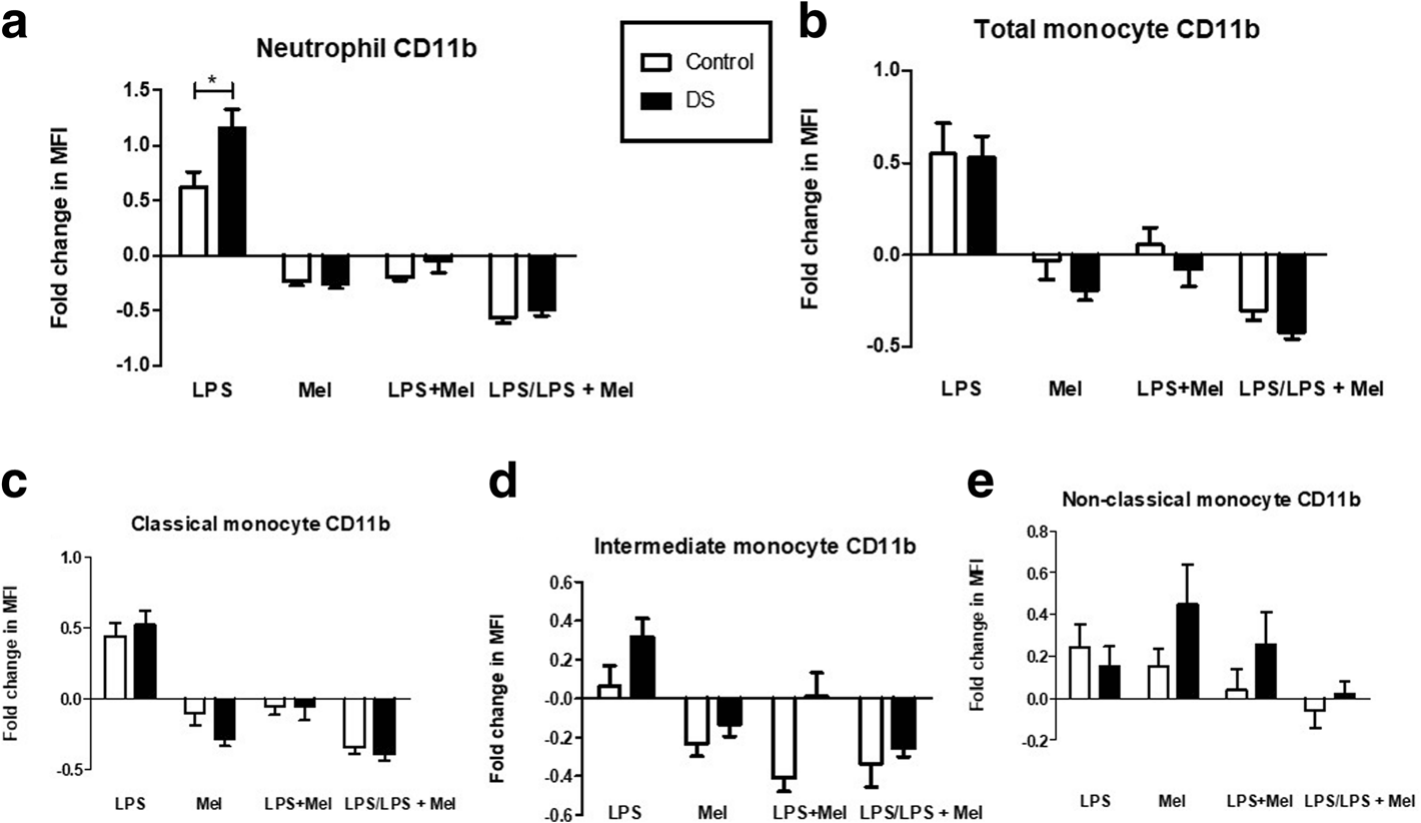
Neutrophil and monocyte CD11b expression in response to LPS and melatonin in children with DS versus controls: Samples were treated with Lipopolysaccharide (LPS), Melatonin (Mel), Lipopolysaccharide and melatonin (LPS + Mel) and Lipopolysaccharide (LPS) versus Lipopolysaccharide and melatonin (LPS/ LPS + Mel) and expressed as fold changes in mean channel fluorescence (MFI). p* < 0.05. **a** Neutrophil CD11b (DS *n* = 23; Controls *n* = 16); **b** Total monocyte CD11b (DS *n* = 19; Controls *n* = 21); **c** Classical monocyte CD11b (DS *n* = 19; Controls *n* = 21); **d** Intermediate CD11b (DS *n* = 18; Controls *n* = 20); **e** Non-classical monocyte CD11b (DS *n* = 19; Controls *n* = 21)

Neutrophil CD11b expression decreased significantly after melatonin treatment in both cohorts (DS *p* = < 0.0001; Controls *p* = < 0.0001), compared with baseline the mean percentage fall in CD11b expression was 25.8% in children with DS versus 23.1% in controls (*p* = 0.63)). There were no differences in mean percentage fall in CD11b expression when comparing LPS treated samples and those treated with LPS and melatonin in both cohorts (Fig. 4a(*p* = 0.64)).

Total monocyte CD11b expression reduced significantly after melatonin incubation in children with DS (*n* = 12; *p* = 0.02), but not in the control group (*n* = 17; *p* = 0.12). The mean percentage fall in CD11b MFI was 19% in children with DS versus 3.4% in controls (Fig. 4b(*p* = 0.24)). In classical and intermediate monocytes there were significant decreases in CD11b expression from baseline after melatonin in both cohorts) DS (*p* = 0.001); Control (*p* = 0.05); (d) DS (*p* = 0.02); Control (*p* = 0.03)]. Non-classical monocytes (CD14dim/CD16-) showed a significant increase in CD11b expression after melatonin in children with DS (*p* = 0.03), and in controls but not to a significant level in the latter (*p* = 0.1). The mean percentage rise in CD11b expression after melatonin was 45% in children with DS versus 15.3% in controls (Fig. 4e (*p* = 0.12)).

### Effects of melatonin on TLR-4 expression

**Fig. 5:**
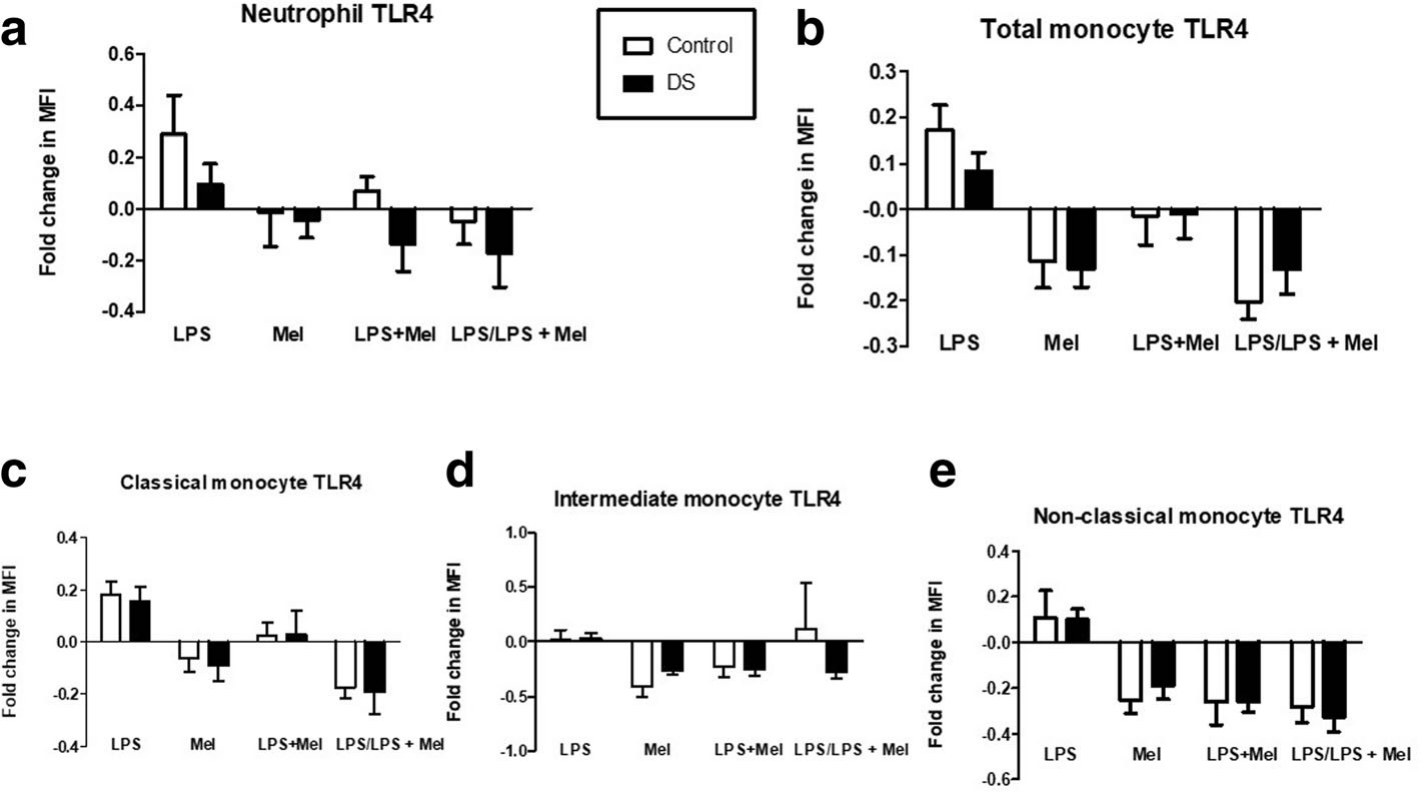
Neutrophil and monocyte TLR-4 expression in response to LPS and melatonin in children with DS versus controls: Samples were treated with Lipopolysaccharide (LPS), Melatonin (Mel), Lipopolysaccharide and melatonin (LPS + Mel) and Lipopolysaccharide (LPS) versus Lipopolysaccharide and melatonin (LPS/ LPS + Mel) and expressed as fold changes in mean channel fluorescence (MFI). **a** Neutrophil TLR-4 (DS *n* = 19; Controls *n* = 10); **b** Total monocyte TLR-4 (DS *n* = 22; Controls *n* = 15); **c** Classical monocyte TLR-4 (DS *n* = 16; Controls *n* = 15); **d** Intermediate monocyte TLR-4 (DS *n* = 15; Controls *n* = 14); **e** Non-classical monocyte TLR-4 (DS *n* = 16; Controls *n* = 20)

Neutrophil TLR-4 expression showed no significant change after melatonin treatment in either group. The mean percentage fall in TLR-4 expression was 4.4% in children with DS and 1.3% in controls (*p* = 0.82). Comparing LPS and LPS + melatonin treated samples there was a 17.5% mean reduction in TLR-4 expression on neutrophils of children with DS compared to a fall of 4.8% in controls (*p* = 0.48), Fig. 5a.

Total monocyte TLR-4 was significantly reduced after melatonin incubation in both groups (DS *p* = 0.03; Controls *p* = 0.05). The average percentage fall in TLR-4 expression after melatonin treatment was 13% in children with DS versus 11.4% in controls (Fig. 5b (*p* = 0.81)). Monocyte subset analysis of melatonin on TLR-4 expression showed no significant reduction in either group (Fig. 5c (*p* = 0.74), d (*p* = 0.23), e (*p* = 0.52)).

## Discussion

Neutrophil CD11b expression at baseline was significantly lower in children with DS compared with controls. Following LPS treatment children with DS upregulated CD11b, and this was significantly greater than controls. Novo et al. [18] reported that, at baseline, CD11b expression on neutrophils was not significantly different between children with DS (*n* = 12) and controls, although the smaller numbers and older population in this study may contribute to these findings. Our research suggests that although the level of CD11b may be lower under normal conditions, after contact with endotoxin there is an increased ability to activate and mobilise neutrophils in response to this stimulus. Neutrophils in children with DS may be hyper-responsive to endotoxin, which may have detrimental effects in the setting of sepsis. Adults with sepsis and renal injury in the absence of hypotension, have been shown to have increased activation of neutrophils with upregulation of CD11b [31], worsening prognosis. Furthermore, neutrophil mediated lung injury in sepsis, and multi-organ dysfunction (MODS) have been associated with increased CD11b expression on these cells [32, 33]. A blockade of this receptor could have potential benefits in these clinical contexts [34]. In paediatric studies LPS hyper-responsiveness has been demonstrated through increased CD11b expression on neutrophils and monocytes of neonates with encephalopathy [35, 36], these infants having developed significant immune dysregulation. Zhou et al. [37] examined TLR4 signalling and the CD11b response on polymorphonuclear cells in mice. The authors concluded that TLR4 mediates CD11b upregulation and is key for PMN activation in response to LPS. Further correlation between CD11b and TLR4 has been described by Guang et al. [38] who reported that CD11b mediates TLR4 signalling and trafficking in a cell specific manner in dendritic cells and macrophages, having a crucial role in balancing the innate and adaptive response to LPS. It appears that the two receptors are inter-linked and have important regulatory roles on one another in initiating the innate immune response.

Zhang et al. [39] demonstrated that mice deficient in CD11b exposed to *Mycobacterium tuberculosis* developed more severe granulomas, higher leucocyte recruitment and elevated pro-inflammatory cytokines. This demonstrates the immunomodulatory effect neutrophil CD11b expression exerts on the host response to infection. A persistent inflammatory response can be seen in autoimmunity and there is a higher prevalence in DS, recent studies suggest that reduced CD11b is associated with chronic inflammation in SLE and lupus nephritis [40, 41]. Neutrophil CD11b is also decreased in septic shock and correlated with poorer outcomes [42, 43]. In this context, the increased incidence of both autoimmunity and sepsis in DS is particularly noteworthy [16, 44]. We demonstrated that melatonin caused a predominant decrease in CD11b expression in both cohorts; Fig. 4. We also showed that children with DS exhibited a hyper-responsive CD11b response to LPS in neutrophils Fig. 4a. In the acute setting of sepsis/SIRS an upregulation of CD11b may be associated with deleterious effects [45], furthermore, a positive correlation between CD11b expression and the degree of systemic inflammation has been described [46], making melatonin a potential adjunct in acute sepsis/SIRS.

The classical monocyte (CD14+/CD16-) accounts for the largest proportion of monocytes (80–85%) and its main functions include antigen presentation and phagocytosis [47]. We found classical monocytes exhibited significantly higher CD11b expression at baseline, and greater fold increases in CD11b after LPS than other monocyte sub-populations in both groups. This sub-group also displayed the largest rise in TLR-4 after LPS compared with other monocyte subpopulations in both cohorts. This suggests that classical monocytes are significantly pro-inflammatory with the largest CD11b and TLR4 response to LPS than any other sub-population. Regarding differential CD11b expression on monocyte subsets Tak et al. [48] reported no significant differences, whereas another study examining differential in vivo activation of monocyte subsets reported the most significant rise in CD11b on the intermediate monocyte [49]. However, these studies [48, 49] characterised CD11b expression after lower doses of LPS with longer incubations in an adult in vivo setting as compared to our study which was undertaken in a paediatric cohort. Monocyte CD11b was highest on classical and intermediate monocytes [50].

Intermediate monocytes (CD14+/CD16+) are elevated in the setting of acute illness such as sepsis in children [51]. In our study, there was a significant rise in CD11b expression after LPS stimulation in children with DS but not in controls on intermediate monocytes. This adds to the evidence that there are hyper-responsive elements to the innate immune system in children with DS. Indeed, intermediate monocytes produce significant quantities of TNF-α once activated [50]. Previous studies have demonstrated elevated levels of TNF-α in patients with DS compared with healthy controls [13] at baseline. Intermediate monocytes demonstrated the greatest TLR-4 at baseline compared with other monocytes in both groups which has also been demonstrated in adults [20].

Non-classical monocytes (CD14dim/16+) have been implicated in both acute and chronic disease and have a pro-inflammatory phenotype with increased production of IL-1β and TNF-α [47]. This monocyte sub-group had significantly lower CD11b and TLR-4 expression in both groups at baseline. Furthermore, non-classical monocytes demonstrated a relative hypo-responsiveness to LPS versus the other sub-populations. Boyette et al. [50] assessed the phenotype, function, and differentiation monocyte subsets, and reported that non-classical monocytes had the lowest CD11b MFI and that there was the smallest response in this subset following TLR-4 stimulation. We found baseline TLR-4 expression was significantly raised in children with DS versus controls. The TLR-4 response plays a significant role in fighting infection but may also be responsible for the dysregulated inflammation seen in septic shock [52]. Williams et al. noted an increased mortality in mice with polymicrobial sepsis who exhibited early up-regulation of TLR-4, and improved survival in those with suppressed TLR gene expression [53]. Suppression of TLR-4 activation, pro-inflammatory cytokine release, and developing endotoxin tolerance is important in limiting the adverse effects of sepsis. Furthermore, a failure of this protective negative feedback process may contribute to increased mortality in sepsis [54].

We demonstrated that melatonin has an anti-inflammatory influence on innate immune function by reducing CD11b expression on neutrophils and total monocytes in children with DS and controls, thereby inhibiting neutrophil and monocyte activation and migration. Although there is a paucity of literature on the effect of melatonin on CD11b, Alvarez-Sanchez et al. reported a reduction in CD11b in melatonin-treated mice [55]. A significant reduction in TLR-4 expression only occurred in total monocyte populations. Melatonin may act as a TLR-4 antagonist and may be modulated via TLR-4 mediated inflammatory genes through molecule myeloid differentiation factor 88 (MyD88)-dependent and TRIF-dependent signalling pathways [56], thereby attenuating inflammation.

Melatonin has beneficial immunomodulatory effects in the setting of sepsis by inhibiting mitochondrial dysfunction and inflammation, reducing nitrosative and oxidative stress [29]. Melatonin has a robust antioxidant or free radical scavenging activity of [57, 58] and melatonin administration also impairs NF-κB transcriptional activity, reducing pro-inflammatory cytokine (IL-1β, TNF-α, IFN-γ) release and inhibiting activation of the NLRP3 inflammasome [59]. Melatonin improved survival and clinical outcomes in neonates versus controls in sepsis [28, 60, 61]. We have demonstrated that the immunomodulatory effects of melatonin in sepsis can also be broadened to include reducing neutrophil and monocyte activation.

Melatonin increased CD11b expression on non-classical monocytes and to a significant level in the children with DS. However, it has been shown that melatonin can have pro-inflammatory actions in response to endotoxaemia. Effenberger et al. [62] reported that melatonin enhanced the general immune response following LPS treatment. Melatonin may have differing actions on distinct cell lines, with the pro-inflammatory non-classical monocyte being preferentially activated. Further evaluation of the immunomodulatory properties of melatonin in children with DS will allow assessment of its potential as a therapeutic agent.

## Conclusion

This research highlights important differences in the innate immunity of children with DS versus age-matched controls. To our knowledge this has not been studied previously in this population. Children with DS have an increased response to LPS in neutrophils and intermediate monocytes, while also having elevated TLR-4 expression on non-classical monocytes compared to controls. These variations may be a contributory factor in a heightened/dysregulated innate immune response, which may have deleterious effects, leading to the worse outcomes seen in sepsis in these children. Lastly, melatonin could represent a useful clinical adjunct in the treatment of sepsis as an immunomodulator and our study suggests its anti-inflammatory effects also influence neutrophil and monocyte function.

## Abbreviations

CHD: Congenital heart disease
DS: Down syndrome
LPS: Lipopolysaccharide
MFI: Mean channel fluorescence
MODS: Multi organ dysfunction syndrome
MyD88: Myeloid differentiation factor 88
NS: Not significant
OLCHC: Our lady’s children’s hospital Crumlin
PBA: PBS containing 1% bovine serum albumin and 0.02% sodium azide
PBS: Phosphate buffered solution
RTIs: Respiratory tract infections
SD: Standard deviation
SEM: Standard error of the mean
SLE: Systemic lupus erythematosus
TLR-4: Toll like receptor 4
